# Complete mitochondrial genome of *Oberea diversipes* (Coleoptera, Cerambycidae)

**DOI:** 10.1080/23802359.2019.1703585

**Published:** 2020-01-08

**Authors:** Lichao Tian, Jinjun Wang

**Affiliations:** a College of Plant Protection, Southwest University, Chongqing, China;; b Chongqing Landscape and Gardening Research Institute, Chongqing, China;; c Chongqing Urban Landscape Engineering Technology Research Center, Chongqing, China

**Keywords:** Cerambycidae, genus *Oberea*, mitochondrial genome, phylogeny

## Abstract

The complete mitochondrial DNA genome of the *Oberea diversipes* was reported. The genome was a circular molecule of 15,499 base pairs (bp), with 13 protein-coding genes, 22 transfer RNA genes, 2 ribosomal RNA genes, and an estimated 830 bp A + T-rich control region. The total base composition of the mitogenome was A–T bias, with 40.9% for A, 37.0% for T, 13.4% for C, and 8.7% for G. The molecular data presented here would be useful for further study of *O. diversipes*.

Considering the diversity of the family Cerambycidae encompassing over 33,000 species (Ślipiński and Escalona 2013), about 40 cerambycids’ complete mitochondrial genome from submitted remains rather limited.

*Oberea diversipes* was restored to specific ranks (Li et al. 2016). It is widely distributed in central, south and southwest China, Laos and Vietnam, and could be harmful to Mullberry et al.

The specimen was collected from Longcanggou (N29°69′, E102°84′), Yingjing county, Ya’an city, Sichuan province. The specimen was deposited in College of Plant Protection, Southwest University with an accession number SWU-COL-160618.

The complete mitogenome of *O. diversipes* (GenBank accession number MN709785) was a circular molecule of 15,499 bp. The total base composition of the mitogenome was A–T bias, with 40.9% for A, 37.0% for T, 13.4% for C, and 8.7% for G. It contained 37 genes including 13 PCGs, 22 tRNAs, and 2 rRNA genes, and a large non-coding region, 830 bp A + T-rich control region. The gene order and orientation of *O. diversipes* were identical to those observed in other cerambycids’ mitogenomes (Song et al. 2015; Fang et al. 2016; Guo et al. 2016). Twenty-three genes were encoded on the majority strand (J strand) and the remains 13 on the minority strand (N strand). All the PCGs began with an ATN start codon, five PCGs (*ND2*, *Cox1*, *Cox2*, *Atp8*, *ND1*) with an ATT; five genes (*Atp6*, *Cox3*, *ND4*, *ND4l*, *Cob*) with an ATG; three genes (*ND3*, *ND5*, *ND6*) with an ATA. Ten PCGs terminated with a TAN (TAA or TAG) stop codon, whereas 3 genes (*Cox2*, *ND5*, *ND4*) terminated with incomplete stop codons (T––). The size of 22 tRNA genes ranged from 63 bp to 72 bp and all the tRNA could be folded in to the typical clover-leaf secondary structure, except for tRNA^Ser^ (AGN) lack of the dihydrouridine (DHU) stem. The 1286 bp lrRNA and 810 bp srRNA were detected in the genome. The 830 bp A + T-rich region (control region) was located between srRNA and tRNA^Ile^.

The phylogenetic analysis was done based on the 13PCGs of 11 available cerambycids’ mitogenomes and four mitogenomes from Chrysomelidae as outgroups. The 13 individual PCGs were aligned CLUSTAL W (Thompson et al. 1994) within MEGA6 (Tamura et al. 2013), and the conserved regions were selected by using the GBlocks 0.91b (Castresana 2000). The aligned data were concatenated with Sequence Matrix v.1.7.8 (Vaidya et al. 2011). Substitution model selection of each PCG was conducted using PartitionFinder v.1.1.1 (Lanfear et al. 2012). Maximum likelihood (ML) using PhyML 3.0 (Guindon et al. 2010).

A phylogenetic tree was constructed based on the ML method. The ML tree (Figure 1) showed that *O. diversipes* was clustered to Cerambycoides and Lamiinae were monophyly with high value support, which agrees with the traditional classification. However, *Sipiniphilus spinicornis* (belonging to subfamily Philinae) and *Obrium* sp. (belonging to subfamily Cerambycinae) were sister groups, which was different from the current classification (Leschen and Beutel 2014, Chapters 2.1–2.4). Therefore, we need more information and analysis in the further study.

**Figure 1.**
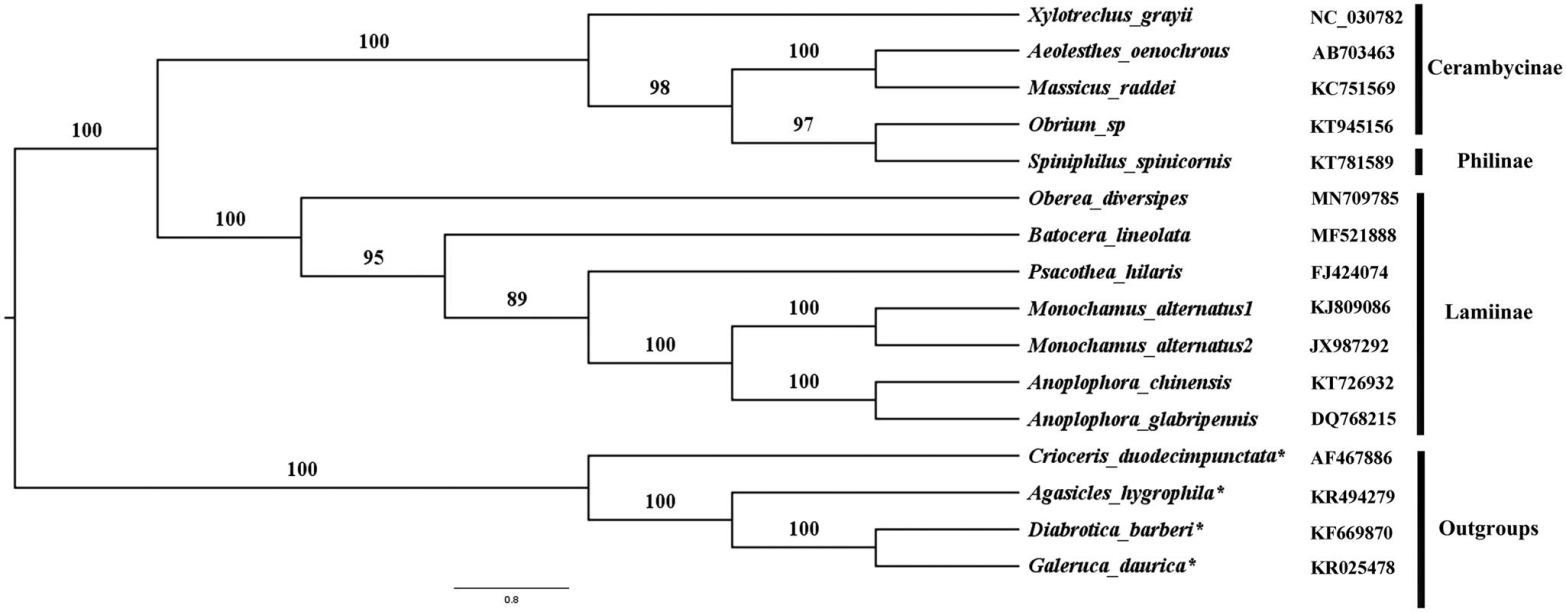
ML tree based on 13 concatenated PCGs data sets.
